# Fusion between M2-macrophages and cancer cells results in a subpopulation of radioresistant cells with enhanced DNA-repair capacity

**DOI:** 10.18632/oncotarget.17986

**Published:** 2017-05-18

**Authors:** Annelie Lindström, Kristine Midtbö, Lars-Gunnar Arnesson, Stina Garvin, Ivan Shabo

**Affiliations:** ^1^ Division of Cell Biology, Department of Clinical and Experimental Medicine, Linköping University, SE 581 85, Linköping, Sweden; ^2^ Department of Clinical Pathology, Department of Clinical and Experimental Medicine, Linköping University, SE 581 85, Linköping, Sweden; ^3^ Division of Surgery, Department of Clinical and Experimental Medicine, Linköping University, SE 581 85, Linköping, Sweden; ^4^ Endocrine and Sarcoma Surgery Unit, Department of Molecular Medicine and Surgery, Karolinska Institutet, SE 171 77, Stockholm, Sweden; ^5^ Department of Breast and Endocrine Surgery, Karolinska University Hospital, SE 171 76, Stockholm, Sweden

**Keywords:** cell fusion, M2-macrophage, radiotherapy, breast cancer, radioresistance

## Abstract

Cell fusion is a natural biological process in normal development and tissue regeneration. Fusion between cancer cells and macrophages results in hybrids that acquire genetic and phenotypic characteristics from both maternal cells. There is a growing body of *in vitro* and *in vivo* data indicating that this process also occurs in solid tumors and may play a significant role in tumor progression. However, investigations of the response of macrophage:cancer cell hybrids to radiotherapy have been lacking. In this study, macrophage:MCF-7 hybrids were generated by spontaneous *in vitro* cell fusion. After irradiation, both hybrids and their maternal MCF-7 cells were treated with 0 Gy, 2.5 Gy and 5 Gy γ-radiation and examined by clonogenic survival and comet assays at three time points (0 h, 24 h, and 48 h). Compared to maternal MCF-7 cells, the hybrids showed increased survival fraction and plating efficiency (colony formation ability) after radiation. The hybrids developed less DNA-damage, expressed significantly lower residual DNA-damage, and after higher radiation dose showed less heterogeneity in DNA-damage compared to their maternal MCF-7 cells. To our knowledge this is the first study that demonstrates that macrophage:cancer cell fusion generates a subpopulation of radioresistant cells with enhanced DNA-repair capacity. These findings provide new insight into how the cell fusion process may contribute to clonal expansion and tumor heterogeneity. Furthermore, our results provide support for cell fusion as a mechanism behind the development of radioresistance and tumor recurrence.

## INTRODUCTION

Accumulating evidence suggests that cell fusion between tumor associated macrophages and cancer cells may be an underlying cause of tumor progression. The fusion process generates hybrids that acquire genetic and phenotypic characteristics from both maternal cells and exhibit a metastatic phenotype [1, 2]. Macrophage traits in tumor cells, such as CD163 expression, are reported in breast cancer and are associated with early tumor recurrence and reduced patient survival [3–5]. Based on cell fusion theory, fusion between tumor-associated macrophages (TAM) and cancer cells yields hybrid cancer cells with macrophage phenotype [6–8]. Spontaneous cell fusion is a complicated cellular process that occurs in solid tumors at low rates, but it is likely to increase under certain conditions such as radiation and inflammation [8–11].

Radiotherapy (RT) kills cancer cells by inducing DNA-damage, either directly or by creating free radicals that in turn damage the DNA. RT also initiates multiple signal transduction cascades regulating apoptosis as well as DNA-repair, dictating the final fate of the cancer cell [12–14]. Breast cancer (BRC) is the most common tumor in females with an incidence of 94/100 000 and a mortality rate of 23/100 000 cases annually [15]. Breast conserving surgery (BCS) in combination with postoperative radiotherapy (RT) is an established treatment of BRC and shown to result in equivalent disease-free and overall survival rates as compared to mastectomy [16]. The purpose of RT is to eliminate microscopic tumor foci in the conserved breast. In spite of radical resection of BRC and postoperative RT, ipsilateral local recurrence (ILR) of the primary tumor occurs in about 10% of females after BCS and is associated with increased risk of distant metastases and poor survival [17–20]. Tumor recurrence is suggested to be caused by the selection of therapy resistant cell clones, which in later stages of disease become a source of tumor recurrence and metastasis [21, 22].

Intra-tumoral heterogeneity is an important phenomenon in tumor biology and generates subpopulations of cancer cells that may be resistant to oncologic treatment and contribute to tumor growth [23–25]. The mechanisms of resistance and clonal heterogeneity in cancer remain enigmatic and pose challenges for oncologic treatment. Accumulating evidence suggests that cell fusion in cancer constitutes a source of heterogeneity and generates treatment-resistant hybrid cells [26–30].

In this study we investigate the role of cell fusion between human M2-macrophages and breast cancer cells in the development of malignant cell clones (macrophage:MCF-7 hybrids) resistant to RT. The study utilizes alkaline single cell gel electrophoresis (SCGE), or comet assay, a reliable method for detecting radiation-induced DNA-damage and repair at a single cell level [31, 32]. Our aim is to increase our understanding of the role of cell fusion in the development of radiation resistance and its possible contribution to tumor recurrence in breast cancer.

## RESULTS

### Generation and definition of hybrids

**Figure 1:**
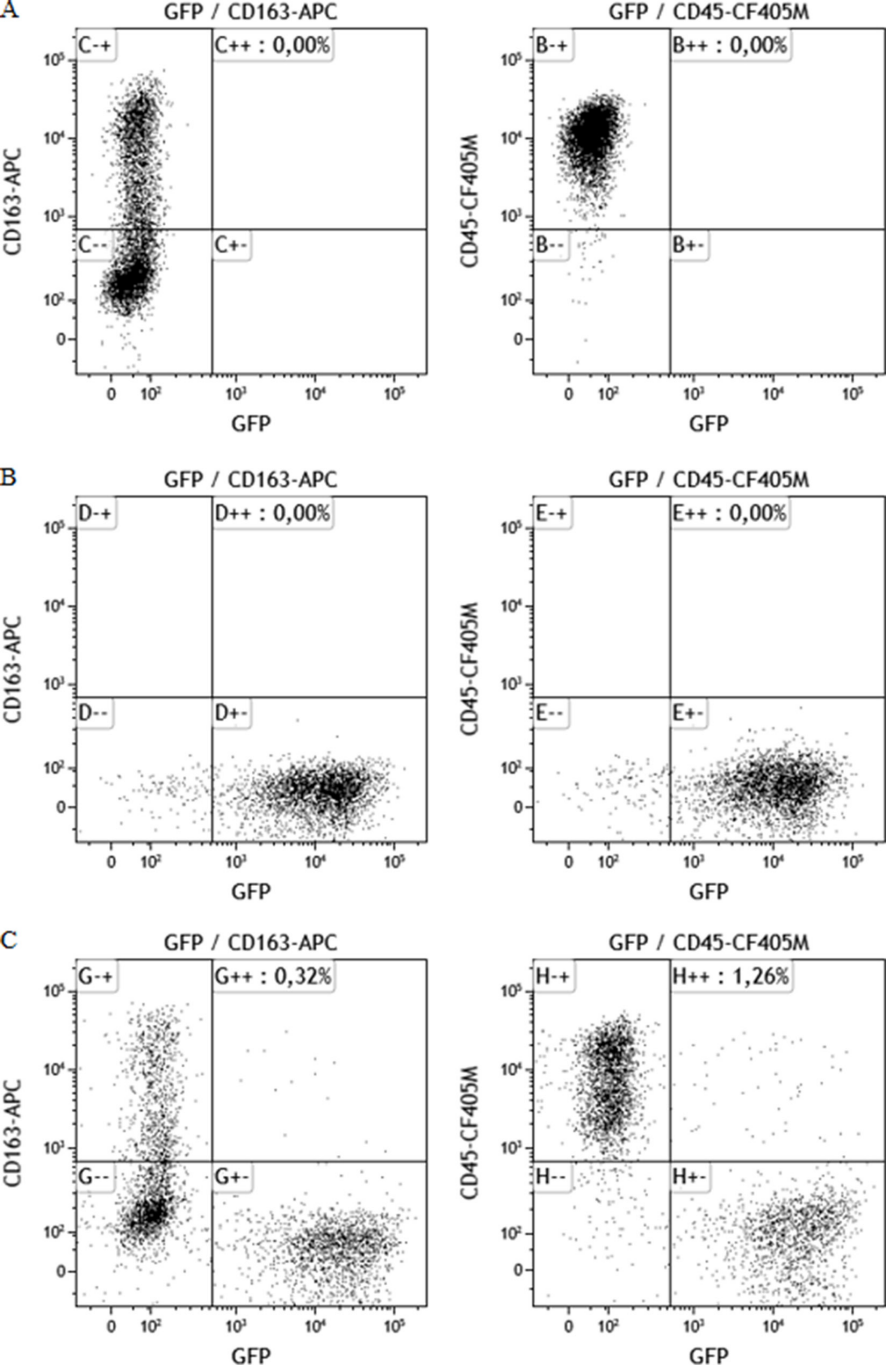
Dot plots showing cellular staining of CD163, CD45 and GFP All plots show events previously gated in a side scatter and forward scatter plot (showing size and granularity of the cells) to ensure populations based on events consisting of single cells only. (**A**) Macrophages cultures show CD45+/CD163+/GFP–, (**B**) MCF-7/GFP cells, transfected with the GFP gene show no positive staining for CD163 or CD45, (**C**) Co-cultures of MCF-7 cells and macrophages yield a MCF-7 population (GFP+), a macrophage population (CD163+/CD45+) and a double positive hybrid population (CD163+/CD45+/GFP+).

Macrophage:MCF-7 hybrids were generated spontaneously by co-culturing MCF-7 cancer cells with M2-differentiated macrophages. GFP-/CD163+/CD45+ cells were defined as M2-macrophages. GFP+/CD163+/CD45+ cells were defined as hybrids and GFP+/CD163–/CD45– cells were defined as MCF-7 cancer cells. Dot plots are demonstrated in Figure 1. The proportion of hybrids harvested by fluorescence-activated cell sorting (FACS) was approximately 2 %.

### Hybrids showed higher cell survival and cloning efficiency following radiation than maternal MCF-7 cells

**Figure 2:**
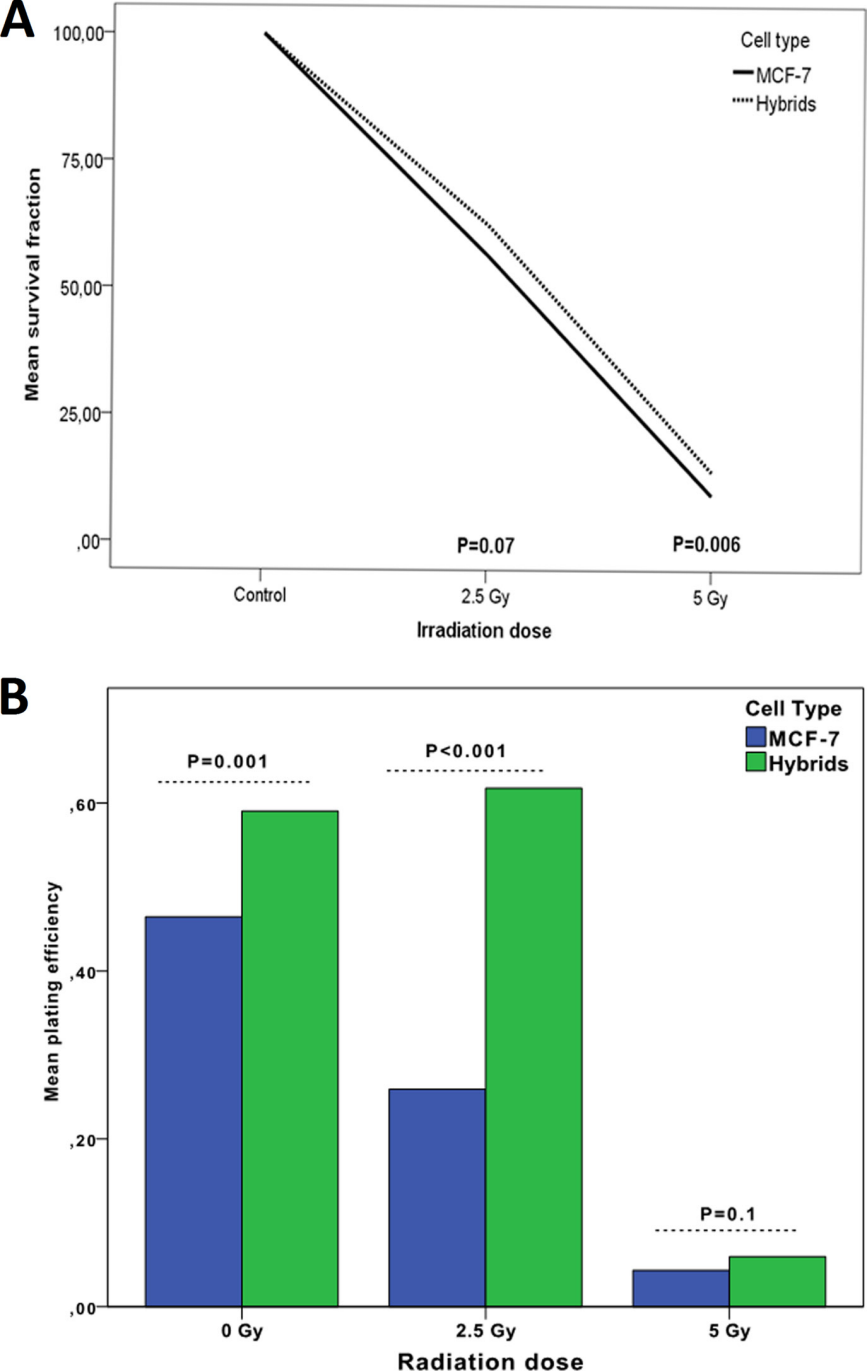
Survival fraction (**A**) and plating efficiency (**B**) of MCF-7 cells compared to macrophage:MCF-7 cell hybrids treated with 0–5 Gy γ-radiation. The 0 Gy value is considered as baseline value (control).

Survival fraction (SF) was investigated by clonogenic assay after radiation doses of 0 Gy (control), 2.5 Gy and 5 Gy. In both MCF-7 cells and hybrids, the SF was inversely proportional to the radiation dose. The mean survival fraction for MCF-7 cells was 52% at 2.5 Gy and 9.5% at 5 Gy while the corresponding results for hybrids were 58% and 14%, respectively. The hybrids showed distinctly higher survival fractions than MCF-7 cells at both 2.5 Gy and 5 Gy radiation, statistically significant at 5 Gy (*p* = 0.006) (Figure 2A).

The plating efficiency (PE) was measured to test colony forming ability of MCF-7 and hybrids after 2.5 Gy and 5 Gy, compared to untreated cells. The mean PE for untreated MCF-7 cells was 46% which was significantly lower compared to the mean PE for hybrids (60%; *p* = 0.001). The mean PE of MCF-7 decreased significantly to 26% and 4% at radiation doses of 2.5 Gy and 5 Gy, respectively. The mean PE for hybrids continued to be high (62%, *p* < 0.001) at radiation dose of 2.5 Gy. Interestingly, the mean PE of MCF-7 and hybrids decreased to similar levels at a radiation dose of 5 Gy; 4% and 6%, respectively (Table 1). There was no significant difference in mean PE between the cells at 5 Gy (Figure 2B).

**Table 1:** Plating efficiency of MCF-7 and macrophage:MCF-7 cell hybrids in relation to radiation

Cell type	Radiation dose	Plating efficiency (%)		
		Mean	SEM	(*p*-value)
MCF7	0 Gy	0.46	0.03	
	2.5 Gy	0.26	0.01	< 0.001
	5 Gy	0.04	0.002	< 0.001
Hybrids	0 Gy	0.6	0.02	
	2.5 Gy	0.6	0.03	1
	5 Gy	0.06	0.01	< 0.001

### Less radiation-induced DNA-damage in hybrids than in maternal MCF-7 cells

**Figure 3:**
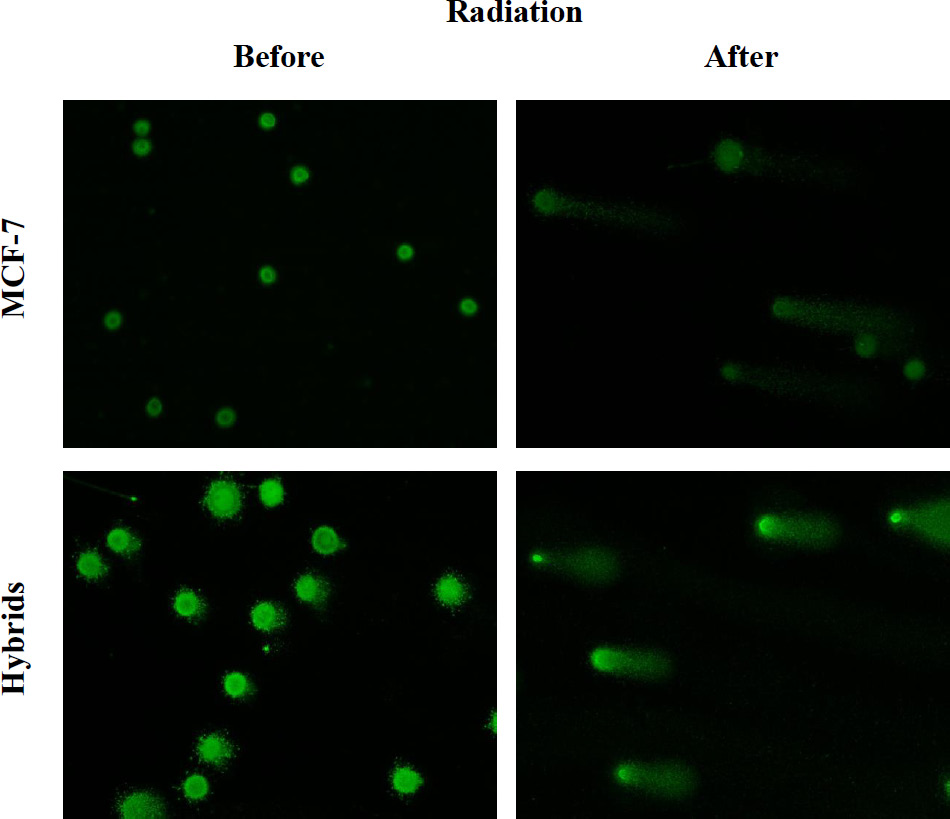
Images after alkaline SCGE demonstrating MCF-7 cells and macrophage:MCF-7 cells hybrids before and after radiation DNA-damage appears as fluorescent comets (tails), originating from cell nucleoids as migrating DNA fragments. Image analysis was performed by ZEN 2.3 software (Bergman Labora Nikon Eclipse E600, Nikon Digital Sight DS-US camera) and NIS Elements BR, Nikon. The images are captured in × 20 magnification.

Extent of radiation-induced DNA-damage was analyzed by SCGE using alkaline electrophoresis, quantified as an increase in tail moment (TM), which is proportional to the number of DNA strand breaks per cell [33]. Representative images of MCF-7 and hybrid comets, evaluated before and after radiation, are shown in Figure 3.

**Figure 4:**
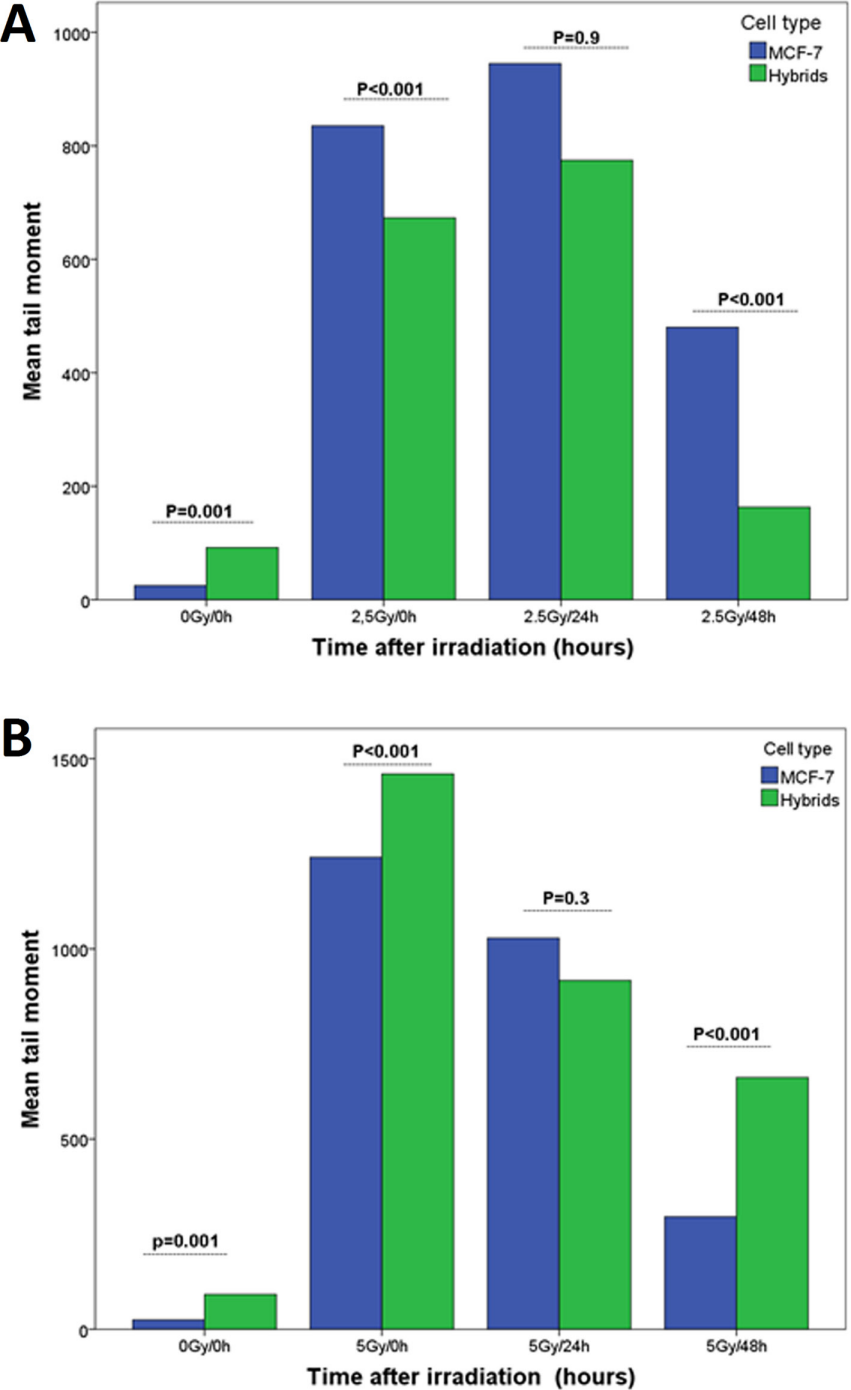
DNA-damage estimated as tail moment (TM) and measured by SCGE performed at three time points (0, 24 and 48 hours) after radiation with (**A**) 2.5 Gy and (**B**) 5 Gy γ-radiation.

Immediately (0h) after 2.5 Gy radiation, the hybrids showed significantly lower DNA-damage (mean TM of 673 ± SEM 47) compared to MCF-7 cells (mean TM of 835 ± SEM 45; *p* < 0.001). However, 5 Gy radiation induced significantly higher mean TM (1460 ± SEM 46) in hybrids compared to MCF-7 cells (1241, ± SEM 79.5), and the comets developed in equal extent in both cell types. Twenty-four hours after 2.5 Gy and 5 Gy radiation, the difference in mean TM between the cell types was not significant (Figure 4). At 48 hours after 2.5 Gy and 5 Gy radiation, the mean TM decreased in both cell types significantly compared to mean TM at 0 and 24 hours (Table 2).

**Table 2:** DNA-damage measured as tail moment (TM) of MCF-7 cells and macrophage:MCF-7 hybrids in relation to 0 Gy (control), 2.5 Gy and 5 Gy radiation doses and post-radiation time (0, 24 and 48 hours)

	Radiation dose (Gy)	Repair time (h)	No. of comets per 150 cell (%)	Tail moment Mean (± SEM)	*P*
MCF-7	0 Gy	control	4 (2.7)	25 (11.6)	
	2.5 Gy	0	150 (100)	835 (45)	
		24	134 (89)	945 (116)	0.55
		48	126 (84)	480 (31)	0.002
					
	5 Gy	0	150 (100)	1241 (79.5)	
		24	150 (100)	1028 (57)	0.024
		48	143 (95)	296 (16)	< 0.001
					
Hybrids	0 Gy	control	47 (31)	92 (15.6)	
	2.5 Gy	0	129 (86)	673 (47)	
		24	145 (97)	774.6 (44)	0.16
		48	86 (57)	163 (19)	< 0.001
					
	5 Gy	0	150 (100)	1460 (46)	
		24	150(100)	917 (45)	< 0.001
		48	150 (100)	661 (29)	< 0.001

### Kinetics of DNA-repair

In order to investigate the kinetics of DNA-repair, the changes in the residual DNA-damage (RDD) in MCF-7 cells and hybrids were calculated 24 h and 48 h after radiation. The results are expressed as %tail DNA of initial DNA-damage in untreated cells (0 Gy and 0 h). The RDD in both MCF-7 cells and hybrids was highest immediately after irradiation (0 h) compared to untreated cells and to treated cells at 24 h and 48 h after radiation. Compared to RDD values 24 h after treatment, the RDD in MCF-7 cells increased after 2.5 Gy and 5 Gy radiation at 48 h. RDD in MCF-7 cells, at 0 h – 48 h, was higher after 5 Gy compared to 2.5 Gy radiation. Hybrids treated with 2.5 Gy radiation expressed significantly higher RDD at 48 h than at 24 h (90% vs 86%; *p* = 0.001). However, interestingly, the RDD in hybrids irradiated with 5 Gy was significantly lower at 48 h than at 24 h after radiation (70% vs 77%; *p* = 0.017) (Table 3).

**Table 3:** Kinetics of DNA-repair in MCF-7 cancer cells and macrophage:MCF-7 hybrids at 24 and 48 hours after 2.5 Gy and 5 Gy radiation dose, respectively

	Radiation dose (Gy)	Repair time (h)	% Tail DNA Mean (± SEM)	% of residual tail DNA	*P*-value
MCF-7	0 Gy	0	0.7 (0.3)		
					
	2.5 Gy	0	11 (0.4)	1571*	< 0.001*
		24	19 (0.9)	90	
		48	21 (1)	105	< 0.001
					
	5 Gy	0	34 (0.8)	4857*	< 0.001*
		24	25 (0.8)	190	
		48	21 (0.9)	205	0.001
					
Hybrids	0 Gy	0	8 (1)		
					
	2.5 Gy	0	18 (0.9)	225*	< 0.001*
		24	22 (1)	86	
		48	18 (0.7)	90	0.001
					
	5 Gy	0	35 (0.9)	437*	< 0.001*
		24	29 (1)	77.5	
		48	27 (0.6)	70	0.017

*P-value for differences between the cells at 2.5 Gy 0 h vs 0 Gy 0 h and at 5 Gy 0 h vs 0 Gy 0 h.

### Heterogeneity in DNA-damage

**Figure 5:**
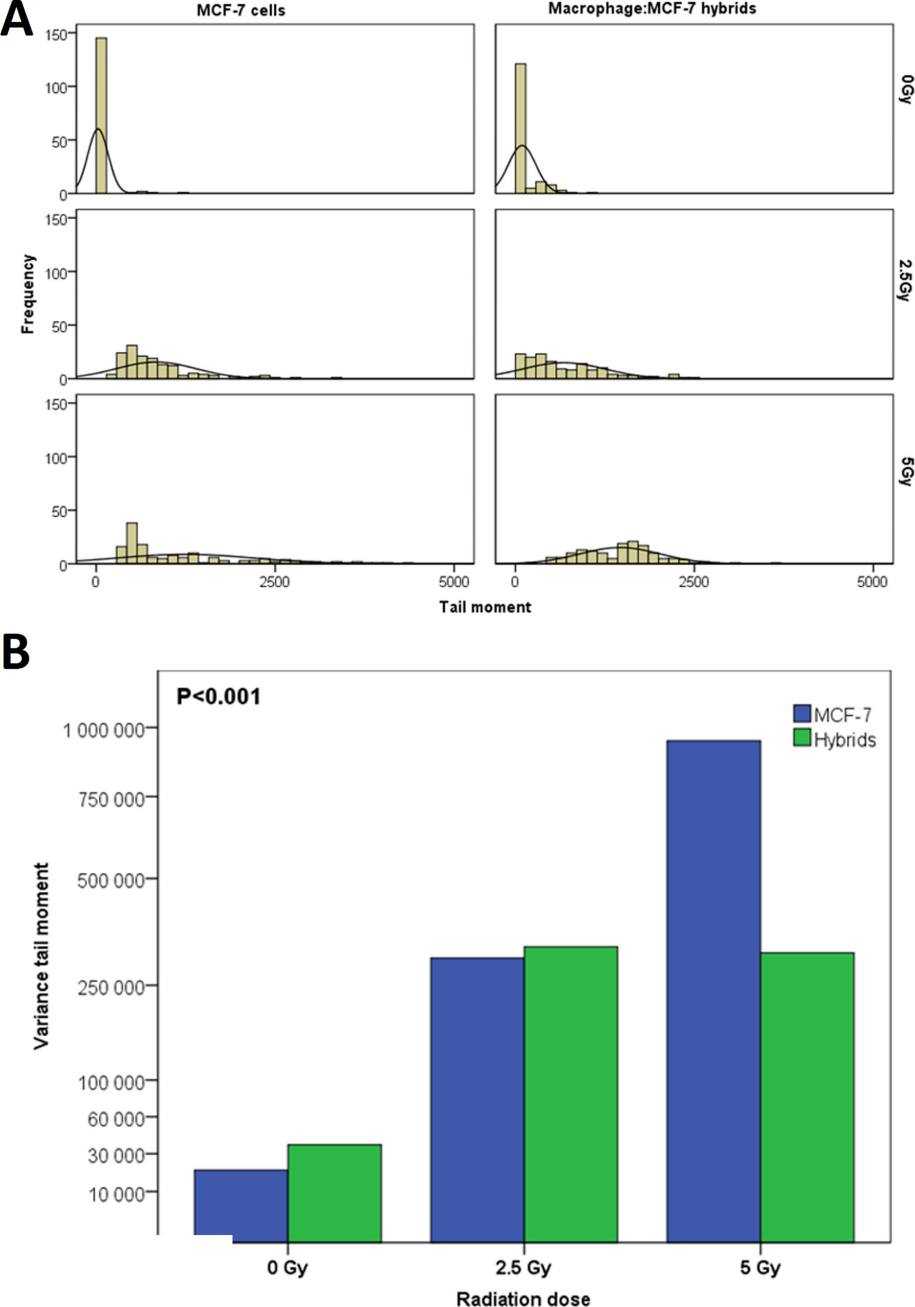
(**A**) The heterogeneity of DNA-damage in MCF-7 cells and macrophage:MCF-7 cells hybrid in relation to γ-radiation (0–5 Gy). (**B**) The variance in DNA-damage for MCF-7 and hybrids increased after radiation. In MCF-7 cells, the variance in DNA-damage was proportional to radiation dose but in hybrids remained unchanged at 2.5 Gy and 5 Gy.

Heterogeneity of DNA-damage was estimated by variance of TM for each radiation dose. The heterogeneity of TM in both MCF-7 and hybrid cells increased significantly immediately after radiation (*p* = 0.001) (Figure 5A). The mean variance of TM in MCF-7 cells after 5 Gy was considerably greater than that after 2.5 Gy, whereas the TM variance in hybrids was similar after 2.5 Gy and 5 Gy. The MCF-7 cells showed significantly higher TM variance compared to hybrids after 5 Gy radiation, but after 2.5 Gy the TM variance was approximately equal in both cell types (Figure 5B).

## DISCUSSION

Clonal evolution in solid tumors contributes to intratumoral heterogeneity and results in the development of subpopulations of cancer cells with different responses to oncological treatment [34–36]. In this study, we demonstrate that fusion between M2-macrophages and MCF-7 breast cancer cells generate hybrid cells that show less DNA-damage, decreased residual DNA-damage, and exhibit extended survival compared to their maternal MCF-7 cancer cells after radiation. The study is based on the SCGE, which is a reliable method that offers a technique for detecting radiation induced DNA damage and repair at single cell level. The advantage of this *in vitro* experimental model is that the effect of radiation on hybrid cells and their maternal cancer cells can be investigated independently of other influencing mechanisms such as paracrine interactions with non-neoplastic cells in tumor microenvironment.

### Cell fusion is associated with resistance to oncologic treatment

Tumor cells are fusogenic and they fuse with other cancer cells and non-neoplastic cells in tumor stroma. Spontaneous fusion between cancer cells is a well documented phenomenon in solid tumors and generates heterogeneous subpopulation of cells that exhibit resistance to chemotherapy [37–40]. Heterotypic cell fusion occurs between cancer cell and bone marrow derived cells, like macrophages, and results in hybrids with increased metastatic features [1, 37–39]. In this study, the hybrids expressed phenotypic traits from both maternal cells, which is consistent with previous observations reported by our group and other *in vitro* and *in vivo* studies [7, 8, 10, 41–43]. The hybrids exhibited higher survival fraction and plating efficiency following radiation compared to maternal MCF-7. Colony formation in cancer cells is an important characteristic for survival and dissemination. DNA-damage is not always lethal to the cell. Rather, tumor cells exposed to DNA-damaging agents may be inactivated [44–46], exhibiting decreased proliferation. In cancer cells, the extent of radiation induced cell division delay is influenced by the cell cycle phase in which the cells are. Cells irradiated in S and G2 phases demonstrate greater division delay than cells in G1. The greater the number of proliferating cells in tumor stroma, the more radiosensitive a tumor will be [47–49]. Moreover, DNA-repair is less efficient during S-phase compared to other phases in cell cycle [50–53]. Several reports indicate that fusion between cancer cells induces cellular senescence and generate slowly growing (dormant) hybrid cells that become non-apoptotic and resistant to chemo- and radiotherapy [54–56]. Hybrids, generated by fusion between cancer cells and bone marrow derived cells, show growth inhibition often in combination with enhanced metastatic properties [57, 58]. Hence, cell fusion may induce growth inhibition with fewer cancer cells in S-phase, contributing to more efficient DNA-repair capacity and less DNA residual damage. These growth arrested cells might remain viable and contribute to cancer recurrence by later generating repopulating tumor cell progeny [59–61]. Taken together, these observations are coherent with the findings presented in this study and suggest that hybrids exposed to radiation might remain viable and retain colony formation ability. Thus, the fusion between macrophages and cancer cells may represent a significant mechanism contributing to the development of treatment resistance in tumor cells.

Another molecular mechanism involved in radiotherapy resistance in hybrid cells may involve regulation of reactive oxygen species (ROS). Ionizing radiation generates ROS, which are reactive with several cellular macromolecules, including DNA, and promote genomic instability by causing accumulation of mutations [62]. ROS-scavengers eliminate ROS and counteract the toxic effects of radiation. Since ROS play an important role in radiation-induced cell death, it is logical to suggest that ROS-scavengers might be involved in hybrid cell response to radiation. In cancer cells with acquired radiation resistance, all classes of ROS-scavengers are up-regulated. On the other hand, downregulation of ROS-scavengers increases the level of ROS and results in improved response of the tumor cells to radiation [63–65]. For example, Ape1/Ref1 is a ROS-scavenger protein that acts as a DNA repair enzyme. In breast and prostate cancer cell lines, overexpression Ape1/Ref1 is associated with restoration of radiation sensitivity and enhanced DNA repair [64, 66, 67]. One hypothesis is therefore that hybrid cells can effectively diminish radiation-induced ROS-formation and ROS-induced DNA damage by activating ROS-scavenging machinery. Reduced intracellular ROS content would decrease radiation-induced DNA damage, enhance DNA repair, and contribute to radiation resistance [68].

### Cell fusion, radiation and DNA-damage

Ionizing radiation has genotoxic effects on cancer cells by directly damaging the molecular structures of DNA and consequently inhibits cell proliferation and induces cell death. DNA-repair pathways recognize and remove the genomic lesions, but unrepaired DNA-damage will in most cases induce cellular senescence or apoptosis [69, 70].

To our knowledge, there are no previous studies investigating how fusion of macrophages and cancer cells impacts the radiation response in solid tumors and more specifically in breast cancer. However, previous reports have shown that cell fusion generates cell clones (hybrids) with reduced susceptibility to other forms of oncological treatments. In an *in vitro* study, Yang et al (2010) reported that cell fusion among MCF-7 cancer cells could be induced by doxorubicin treatment and that MCF-7 hybrid cells acquired a doxorubicin-resistant phenotype [37]. Wang et al (2012) showed that spontaneous fusion between RL-1 prostate cancer cells and HPS-15 stroma cells generated subpopulations of malignant cells that exhibited sustained androgen receptor expression during androgen deprivation and increased levels of prostate specific antigen (PSA) indicating androgen insensitivity and androgen independence [71]. Moreover, in an *in vitro* experiment, Kaur et al (2015) reported a high frequency of homotypic cell fusion in a glioblastoma cell line resulting in a hybrid subpopulation demonstrating decreased radiation-induced stress, increased senescence-associated secretory proteins (SASPs), and upregulated anti-apoptotic genes like BIRC3 and Bcl-xL [55]. In the current study, the hybrids acquired significantly less DNA-damage after 2.5 Gy but similar levels of DNA-damage after 5 Gy radiation as compared to their maternal MCF-7 cells. These data suggest that higher radiation doses may be required to achieve genotoxic effects in hybrid cells.

### Kinetics of DNA-repair

DNA-repair capacity is a predisposing factor in cancer susceptibility and influences radiosensitivity [72–75]. Compared to maternal MCF-7 cells, hybrids showed significantly lower residual DNA-damage immediately after radiation as well as 24 and 48 hours after exposure. In addition, the hybrids demonstrated significantly higher survival fractions than MCF-7 cells, especially after 5 Gy radiation dose. Taken together, these findings suggest that hybrids acquire enhanced DNA-damage recovery capacity and decreased radiosensitivity.

### Radiation damage heterogeneity

Intratumoral heterogeneity is a hallmark of solid tumors, thought to arise through a complex evolution of genetic, epigenetic, and functional diversity in tumor cells as they interact in the tumor microenvironment [76–79]. Somatic cell fusion causes nuclear reprogramming with genetic and phenotypic diversity in tumors, which in turn contributes to the development of tumor heterogeneity [80, 81]. In this paper, MCF-7 and hybrids showed significant heterogeneity in radiation induced-DNA-damage. While the variance of DNA-damages in MCF-7 cells was proportional to the radiation dose (0 – 5 Gy), the hybrids showed no significant difference in DNA-damage variance between 2.5 and 5 Gy. Thus, DNA damage heterogeneity in hybrids is not proportional to radiation at doses of 2.5 – 5 Gy, suggesting that hybrids might have greater genetic stability than MCF-7 cells.

### Cell fusion in human cancers and its clinical significance

Cell fusion is a natural biological process in normal development and tissue regeneration. *In vitro* and *in vivo* experimental data indicate that cell fusion occurs in solid tumors and may play a significant role in clinical tumor progression [8, 82–85]. Silk et al (2013) provided clinical evidence for cell fusion between transplanted bone marrow derived cells and human intestinal epithelium [6]. In human cancers, it is difficult to confirm cell fusion because of the shared genetic content of macrophages, cancer cells, and hybrids and the lack of tissue specific markers indicative of fusion between the two cell types. However, by studying transplant patients it is possible to distinguish the genetic sources of the tumor cells and clinical evidence indicating leucocyte-tumor cell hybridization in human cancers has been demonstrated in several case reports. Chakraborty et al (2004) and Yilmaz et al (2005) both reported that in renal cell carcinoma patients who had previously received allogeneic bone marrow transplants (BMT), alleles from both donor and recipient were found in primary tumor cells, indicating that fusion between tumor cells and donor bone marrow derived cells had occurred [86, 87]. Lazova et al (2011) analyzed the genotype of a melanoma brain metastasis from a patient who had previously received an allogeneic BMT and found that tumor cells contained alleles from donor and patient pre-BMT lymphocytes with similar allelic ratios, suggesting that the tumor was generated from a single fusion event or hybrid cell clone [88]. In another case, a patient who received an allogeneic BMT and later developed malignant melanoma, the melanoma cells within the lymph node metastasis contained a mixture of the donor-patient genome, again suggesting hybridization between donor bone marrow derived cells and patient tumor cells [89]. These observations constitute clinical evidence for leucocyte-cancer cell hybrid formation in human cancers.

Based on the cell fusion theory and the assumption that macrophage–cancer cell fusion creates hybrids expressing phenotypic characteristics of macrophages, we used the macrophage-specific marker CD163 as a surrogate marker for detecting fusion events in a clinical tumor material. CD163 was expressed by tumor cells in 48% of breast and 20% of colorectal cancer patients and was significantly associated with advanced tumor stages and poor survival [3, 4, 90]. In rectal cancer patients, CD163 expression in tumor cells was found in 17% of primary tumors from patients not treated with preoperative irradiation compared with 31% of those given preoperative radiotherapy (p < 0.044). Moreover, CD163 expression was inversely correlated to apoptosis. In tumors from patients treated with preoperative radiotherapy, apoptosis was significantly higher in CD163-negative tumors. There was no correlation between CD163 expression and apoptosis in tumors from patients who did not receive preoperative radiotherapy [4]. These observations from clinical human tumor material suggest that cell fusion may influence tumor biology including response to radiotherapy.

## MATERIALS AND METHODS

### Cell culture

MCF-7/GFP (AKR-211) breast cancer cell line (Cell Biolabs, INC. San Diego, USA) was cultured in Roswell Park Memorial Institute (RPMI) 1640 medium supplemented with 1% penicillin-streptomycin (PEST) (Thermo Fisher Scientific, USA), 10% FBS, 2.5% HEPES and GlutaMax (Gibco^®^, Life Technologies, USA) in T-75 tissue culture flasks (431464, Corning Incorporated, Sigma-Aldrich Co, ST. Louis, USA) and incubated at 37°C in humidified air 5% CO_2_ atmosphere. Cell medium was changed every 2–3 days, and the cells were passaged with 0.25% trypsin (Gibco^®^, Life Technologies, USA) at 95% confluence.

### Monocyte isolation

Monocytes were isolated from buffy coat obtained from male healthy blood donors at the department of Transfusion Medicine, County Council of Östergötland, in Linköping and Jönköping, Sweden. All the blood donors had given their informed consent according to the local guidelines and the Swedish National Law on ethical review of research involving humans (2003:460: 3–4 §). The buffy coat was mixed with 70 ml NaCl, layered onto 20 ml Lymphoprep (Thermo Fisher Scientific, Waltham, MA USA) in 50 ml centrifuge tubes (91050, Techno Plastic Products, Switzerland) and centrifuged (without brake) at 480 g (Sigma Laboratory 4k15) in room temperature for 40 minutes. The buffy coat layer, generated on top of the Lymphoprep layer, was transferred into new 50 ml tubes containing PBS-Heparin (Medicago Leo Pharma, Denmark) and filled to total volume of 50 ml with PBS-Heparin and centrifuged at 300 g for 10 minutes at 4°C. The cell pellets were washed twice in PBS-Heparin, centrifuged at 220 g for 5 minutes at 4°C, followed by three washing procedures in Krebs-Ringer Bicarbonate Buffer (consisting of 120 mM NaCl, 4.9 mM KCl, 1.2 mM MgSO_4_x7H_2_O, 1.7 mM KH_2_PO_4_x2H_2_O and 10 mM Glucose) without Ca^2+^ and centrifuged at 220 g for 5 minutes at 4°C. The white blood cells were resuspended in 20 ml RPMI1640 medium supplemented with 1% PEST. The number of cells was established using a TC10^TM^ Automated Cell Counter (Bio-Rad Laboratories AB, Solna, Sweden) and seeded into T-75 tissue culture flasks (431464, Corning Incorporated, Sigma-Aldrich Co, ST. Louis, USA) with RPMI 1640 medium (10 ml total volume), supplemented with 1% PEST, and incubated for 1–2 h at 37°C with 5% CO_2_ to allow monocyte adhesion. The non-adherent cells were eliminated by washing 2–3 times using PBS 37°C. The adherent monocytes were allowed to differentiate to macrophages due to incubation (at 37°C in 5% CO_2_) with 40 ng/ml of macrophage colony-stimulating factor, M-CSF (Nordic Biosite, Sweden), for 5–7 days. Induction of M2 polarization of macrophages, the M-CSF differentiated macrophages were stimulated with 20 ng/ml human interleukin-4 (IL-4) (Nordic Biosite, Sweden) for 18–24 h at 37°C and 5% CO_2_.

### Generating and isolation of M2-macrophage:MCF-7 hybrids

Spontaneous cell fusion between M2-macrophages and GFP-labeled MCF-7 cancer cells was induced by co-culturing these cells in the same cell culture vial (T-25 and/or T-75, 430168/431464, culture flasks, Corning Incorporated, Sigma-Aldrich Co, ST. Louis, USA) in RPMI 1640 medium, 37°C in 5% CO_2_, during 2–3 days. The cells were seeded at a ratio of about 3–5:1 (M2-macrophages: MCF-7). After hybridization, cells were harvested with a 0.05% trypsin-EDTA solution (Gibco^®^, Life Technologies, USA), centrifuged at 300g for 5 minutes at 4°C in 1,5 ml eppendorf tubes (Eppendorf) washed with 1 ml PBS 4°C, and resuspended in 95 μl Cell Staining Buffer (Nordic Biosite, Sweden) at a concentration of about 5 × 10^6^ cells/ml. The cell suspension was incubated on ice for 10 min with 5 μl TrueStain FcX solution (BioLegend, San Diego, USA) per 1×10^6^ cells. Combinations of direct conjugated monoclonal anti-human CD163 (APC Anti-human CD163 (IgG1 k), clone GHI/61, 100 μg/ml) and anti-human CD45 (CF405M anti-human CD45 (IgG1 k), clone HI30, 50 μg/ml) antibodies or their respective isotype controls (APC and CF405M mouse IgG1 k, clone MOPC-21, 200 μg/ml) (all antibodies from Biolegend, San Diego, USA) were added to the cell suspension at concentrations recommended by the manufacturer and incubated at 4°C for 30 min in darkness. The samples were centrifuged at 300 g for 5 minutes at 4°C and excess of antibodies was removed with supernatant. The labeled cells were washed twice in 1 ml Cell Staining Buffer/centrifuged at 300 g for 5 minutes at 4 °C, diluted in 1 ml PBS and filtrated in pre-separation filter (30 μm, Miltenyi Biotech, Lund, Sweden) before they were sorted with BD FACSAria™ III (BD Bioscience, USA) (violet laser 405 nm, blue laser 488 nm, green laser 561 nm, red laser 632 nm). The cells were examined in relation to GFP, CD163 and CD45 expression and initially sorted by GFP expression (positive selection of MCF-7/GFP origin) and subsequently by CD163 and CD45 expression. Macrophage:MCF-7 hybrids expressed both GFP and macrophage marker (CD163 and CD45) and cells positive for these markers were collected in tubes (352063, BD Falcon^TM^, Thermo Fisher Scientific) containing 0.5 ml FBS at 4°C.

### Radiation

MCF-7 cells and M2-macrophage:MCF-7 hybrids (5 × 10^5^ cells) were cultured in T-25 tissue (430168, Corning Incorporated, Sigma-Aldrich Co, ST. Louis, USA) culture flasks in complemented RPMI 1640 medium, allowed to grow for 2 days (90–95% confluency). At day 3 after seeding, the cells were exposed to gamma, γ, radiation (Clinac 600C/D, Varian Medical Systems Incorporated, Herlev, Denmark, one AP field, linear accelerated 6MV Photons) with dose rate 5 Gy/minute and doses of 0 (control), 2.5 and 5.0 Gy at room temperature. The culture flasks were surrounded with 3 cm poly methyl methacrylate (PMMA) with density in comparison with human tissue. For the evaluation of initial damage, immediately after irradiation, the samples were kept on ice to avoid repair of irradiation induced damage (0 h, no incubation period). For repair evaluation and view cell recovery from the effects of radiation over time, the cells were incubated at 37°C in humidified air 5% CO_2_ atmosphere for 24 and 48 hours, respectively, after irradiation and prior to examination with alkaline single cell gel electrophoresis (SCGE) and clonogenic assays.

### Alkaline single cell gel electrophoresis (SCGE)

The alkaline single cell gel electrophoresis (SCGE), also called comet assay, is an established technique for quantifying DNA damage in individual cells after irradiation [31, 32]. Single cell suspensions (5–10 μl, 1–1.5 × 10^6^ cells/ml) were mixed with 60–100 μl 0.5% low melting point agarose gel (SeaPlaque^TM^ Agarose, FMC BioProducts, Belgium) and mounted in circles on scratched microscopic slides (76×26x1.1 mm, 201307, RS France, Laboandco Europe, France)(in triplicate). The slides were incubated for 2 minutes at 4°C for gelling and thereafter placed in cold freshly made lysis buffer pH 10 containing 2.5 M NaCl (106404, Merck, Solna, Sweden), 100 mM EDTA (UltraPure^TM^, 15576–028, invitrogen, UK), 10 mM Tris (UltraPure, 819623, ICN Biomedicals, Ohio, USA), and 1% Triton X-100 (136597, Fisher Scientific, USA) for a minimum of 18 h at 4°C.

The slides were incubated in electrophoresis buffer pH > 13 (300 mM NaOH (EKA Nobel), 1 mM EDTA (UltraPure^TM^, 15576–028, Invitrogen, UK)) 20 minutes, at 4°C, and thereafter placed in electric field (4V, 7mA, Power Supply Amersham Pharmacia Biotech, Uppsala, Sweden) using an electrophoresis chamber (Sub-Cell GT Mini, 3866, BioRad, Italy). Electrophoresis was run for 20 min at 4°C. Neutralization was done using drops of neutralization buffer pH 7.5 (0.4 M Tris-HCl, Kebo Lab, Fischer Scientific, USA) three times with 5 minutes interval at 4°C. The slides were incubated in 95% ethanol (0306046346, Kemetyl AB, Haninge, Sweden) for 5 minutes at 4°C, dried in air and stored at room temperature in darkness until stained and analyzed.

For analysis, 100 μl SyberGold staining solution (1 μl SYBER Gold stain 10000x concentrate in DMSO, excitation wavelength 496 nm, max emission wavelength 522 nm, 417851, Invitrogen, Oregon, USA) was diluted into 30 ml TE Buffer (10 mM Tris-HCL from Kebo Lab, Fischer Scientific, USA, and 1 mM EDTA, pH7.5-8.0). In each experiment, a minimum of 150 cells were examined for each radiation dose and 3 independent assays for each cell type. Image analysis was performed by ZEN 2.3 software (Bergman Labora Nikon Eclipse E600, 6 filters, Nikon Digital Sight DS-US camera) [91, 92]. For the evaluation of DNA damage, nucleus intensity, tail intensity and tail length were measured (NIS Elements BR, 4.20.01 64-bit, Nikon). The percentage of tail DNA and tail moment were calculated as the following equations [93]:

Tail % DNA (TD) = 100 × tail intensity / (nucleus intensity + tail intensity)

Tail moment = tail length × tail % DNA

Four experimental time points were evaluated to characterize cellular radiation effects: baseline DNA damage in cells that had not been irradiated 0 Gy 0 h (TD0), and induced DNA damage detected immediately (TDt0), at 24 h (TDt1) and 48 h (TDt2) after irradiation. Residual DNA damage (RDD) at 24 h and 48 h after radiation was calculated according to the following equation:

% Residual DNA damage (RDD t1;t2) = [(TD t1;t2 − TDt0)/ (TDt0 - TD0)] ×100

### Clonogenic assay

After radiation procedure and storage at 4°C, the cells were trypsinated and resuspended in complemented RPMI medium. Cell counts were determined from two aliquots (TC10^TM^ Automated Cell Counter, Bio-Rad Laboratories AB, Solna, Sweden) and mean was used to transfer 10 000 cells into 1.5 ml Eppendorf tubes (E161680L, Eppendorf AG, Hamburg, Germany) and volume adjusted to 1 ml with complemented RPMI medium. Triplicates for each dose were made according to: 10 μl cell suspension (corresponding to 100 cells) was transferred to respectively three 60 mm petri dishes (150288, Nunc^TM^, Thermo Fischer Scientific, Roskilde, Denmark); 20 μl (corresponding to 200 cells) was transferred to respectively three 60 mm petri dishes. Dishes were incubated at with 4 ml complemented RPMI medium at 37°C in humidified air 5% CO_2_ atmosphere, for six days. Dishes were washed with Phosphate Buffer Saline (PBS, 09-2051-100, Medicago, Uppsala, Sweden) and incubated for 30 minutes with 6% glutaraldehyde (Fisher Scientific GTF), 0.5% Crystal Violet (Serva Electrophoresis GmbH, Heidelberg, Germany) fixation/staining solution. The dishes were washed with water and allowed to dry at room temperature and in darkness. Colonies (> 50 cells/colony) were counted using a visible light source (Olympus CH-2, Japan). Plating efficiency (PE) was defined as the proportion of colonies developed from the seeded cells and calculated according to the equation: PE = number of colonies/number of seeded cells. The survival fraction (SF) was calculated as follows: SF = number of colonies formed after irradiation/(number of seeded cells × PE/100) [94, 95].

### Definitions and statistical analysis

Statistical analyses were performed using SPSS statistics software, version 23 (IBM Corporation, USA). Since the results of clonogenic and comet assays were not normally distributed, comparison among cell types, irradiation doses (0 Gy, 2.5 Gy and 5 Gy) and recovery time (0 h, 24 h and 48 h) was carried out by one-way analysis of variance (ANOVA). Heterogeneity of DNA damage was evaluated by variance of DNA damage in tail moment determined for each radiation dose. Non-parametric Mann-Whitney *U*-test was used to test the statistical significance of differences between means of survival fraction, mean variance, plating efficiency, percentage tail DNA, tail moment and residual DNA damage in relation cell types, irradiation doses and recovery time. *P* value < 5% was considered as statistically significant.

## CONCLUSIONS

Hybrid cells that arise after fusion between M2-macrophages and cancer cells acquire decreased radiation sensitivity, show higher DNA-repair capacity, and survive radiation better compared with their maternal cancer cells. To our knowledge, this is the first *in vitro* model showing that cell fusion may contribute to the development of subpopulations of radioresistant cancer cells. These findings provide new insights about the biological significance of cell fusion and how it might contribute to clonal evolution and the development of radiation resistance in solid tumors.
